# Development of a Gene-Centered SSR Atlas as a Resource for Papaya (*Carica papaya*) Marker-Assisted Selection and Population Genetic Studies

**DOI:** 10.1371/journal.pone.0112654

**Published:** 2014-11-13

**Authors:** Newton Medeiros Vidal, Ana Laura Grazziotin, Helaine Christine Cancela Ramos, Messias Gonzaga Pereira, Thiago Motta Venancio

**Affiliations:** 1 Laboratório de Química e Função de Proteínas e Peptídeos, Centro de Biociências e Biotecnologia, Universidade Estadual do Norte Fluminense Darcy Ribeiro, Campos dos Goytacazes, Rio de Janeiro, Brazil; 2 Laboratório de Melhoramento Genético Vegetal, Centro de Ciências e Tecnologias Agropecuárias, Universidade Estadual do Norte Fluminense Darcy Ribeiro, Campos dos Goytacazes, Rio de Janeiro, Brazil; USDA/ARS, United States of America

## Abstract

*Carica papaya* (papaya) is an economically important tropical fruit. Molecular marker-assisted selection is an inexpensive and reliable tool that has been widely used to improve fruit quality traits and resistance against diseases. In the present study we report the development and validation of an atlas of papaya simple sequence repeat (SSR) markers. We integrated gene predictions and functional annotations to provide a gene-centered perspective for marker-assisted selection studies. Our atlas comprises 160,318 SSRs, from which 21,231 were located in genic regions (i.e. inside exons, exon-intron junctions or introns). A total of 116,453 (72.6%) of all identified repeats were successfully mapped to one of the nine papaya linkage groups. Primer pairs were designed for markers from 9,594 genes (34.5% of the papaya gene complement). Using papaya-tomato orthology assessments, we assembled a list of 300 genes (comprising 785 SSRs) potentially involved in fruit ripening. We validated our atlas by screening 73 SSR markers (including 25 fruit ripening genes), achieving 100% amplification rate and uncovering 26% polymorphism rate between the parental genotypes (Sekati and JS12). The SSR atlas presented here is the first comprehensive gene-centered collection of annotated and genome positioned papaya SSRs. These features combined with thousands of high-quality primer pairs make the atlas an important resource for the papaya research community.

## Introduction

Papaya (*Carica papaya* Linneaus) is an economically and nutritionally important fruit tree of tropical and subtropical regions. Papaya is well known for its nutritional benefits [1], medical [2] and industrial [3] applications. Due to its commercial importance, papaya production is currently ranked as the third major global production among tropical fruits [4]. Notwithstanding the increased papaya trade, a limited number of cultivars are commercially available, hampering papaya production worldwide. Further, the low genetic diversity of selected *C. papaya* cultivars [5]–[7] makes the species susceptible to bacterial and viral infections [8], [9]. To improve disease resistance, genetic diversity and productiveness, researchers have been using molecular marker-assisted selection (MAS), a well-established procedure employed in commercial breeding programs to enhance the gain from artificial selection.

Microsatellites, also known as simple sequence repeats (SSRs), are simple tandemly repeated DNA sequences, ranging from 2–6 base pairs per repeat unit [10]. These repeated sequences are highly variable in length, mainly due to unequal recombination events or DNA polymerase slippage. Microsatellite PCR amplification has been described as a reliable, rapid, and inexpensive technique. Combined with the highly polymorphic nature and co-dominant segregation of SSRs, PCR amplification of microsatellites is a powerful technique for plant breeding and genetic studies, such as MAS, population genetic analysis, quantitative trait locus (QTLs) mapping, DNA fingerprinting, and genome mapping [11]–[16].

Once a labor-intensive and time-consuming process, identification of new microsatellite markers became increasingly feasible with the improvement of molecular biology techniques and availability of genomic information for several plant species. Over the past decade, SSRs derived from expressed sequence tags (EST-SSRs) markers emerged as a feasible alternative in marker development for several crop species [17]. EST-SSRs are transcribed from coding sequences (CDS), which tend to be conserved between species, high interspecies transferability rates can be achieved [18], [19]. Moreover, CDS markers are more informative than intergenic ‘anonymous’ markers because they are more likely to be functional [20], [21]. However, there are also a few disadvantages of using CDS markers. High conservation may result in low polymorphism rates, which are of limited use in MAS studies. In addition, primers designed for exon-intron junctions may result in PCR amplification failures.

With the increasing number of sequenced genomes, SSRs can be computationally detected and classified according to their genomic locations in 5′ untranslated regions (UTRs), exons, introns and 3′ UTRs. By using this strategy, exon-intron junctions can be avoided during primer design and fully exonic markers can be selected. Completely sequenced genomes also allow the selection of intronic markers which are more polymorphic than exonic markers and segregate with a particular gene that may be associated with a biochemical function or phenotype of interest [22], [23].

In the present work we describe the analysis of papaya SSR markers in a genome-wide scale, integrating SSR positioning and functional annotation data. Stringent primer design criteria were used to allow better results in genetic studies. This complete catalog will be of great value for the papaya research community, especially for groups conducting MAS projects. Using this map, researchers will be able to filter and choose interesting markers according to SSR type, length, sequence, region location (exon, intron or intergenic), linkage group and gene annotation.

## Materials and Methods

### Genomic data and SSR annotation

Papaya genome assembly and annotation files were downloaded from Phytozome v7.0 FTP (http://www.phytozome.net/) [24]. The genome assembly 113 consists of 244.5 Mb of gapless sequences, distributed in 3,207 scaffolds and 2,693 contigs. Genomic locations of detected SSRs were integrated with gene and exon coordinates from the reference GFF file. Based on their genomic mapping, SSRs were categorized as exonic (entirely within the CDS), exon-intron (in exon-intron boundaries), intronic (within introns) and intergenic (outside of genic regions). Identifiers and annotations based on papaya-*Arabidopsis thaliana* homology were obtained for genic SSRs. Papaya genes were also annotated using Gene Ontology (GO) terms from the Plant Ontology Tool (http://www.arabidopsis.org/tools/bulk/po/index.jsp). In order to map scaffolds and contigs to the major nine linkage groups, all 47,483 papaya contigs [GenBank: ABIM01000001-ABIM01047483] were downloaded from the National Center for Biotechnology Information (http://www.ncbi.nlm.nih.gov). Linkage group information was retrieved from GenBank files for each contig and scaffold [GenBank: DS981520-DS984726].

### Simple Sequence Repeats Identification

Exact maximal repeats were detected in the papaya genome using *mreps* [25]. Perfect repeats with more than 12 nucleotides, motif lengths of 2–6 bp and at least 2 units of repetition were analyzed. Parameters were set as follows: *-r 0 -minsize 12 -minperiod 2 -maxperiod 6 -exp 2*. The *mreps* algorithm finds exact maximal repeats, removes redundancy by selecting the best period for each repeat, merges repeats with same period and eliminates statistically insignificant expected repeats [25].

For description of di- and trinucleotide motifs, circular permutations and complementary strand nucleotides were considered as equivalents and grouped in one class after determining the individual repeat frequencies. Thus, there are four possible di-nucleotide motifs and ten possible tri-nucleotide motifs. For example, motifs AG, GA, CT and TC are equivalent and grouped as AG/GA/CT/TC. Likewise, motifs ACG, CGA, GAC, CGT, TCG and GTC are also equivalents and represented as ACG/CGA/GAC/CGT/TCG/GTC.

### Primer design

SSRs were retrieved from the genome with 250 bp upstream/downstream flanking regions and had their low complexity regions masked with DustMasker [26]. Primers were designed with the standalone version of Primer3 [27] using the following parameters: primer length between 18 and 25 nucleotides (optimal length = 20 nt), melting temperature between 57 and 63°C (optimal Tm = 60°C), PCR product size between 250 and 350 bp (optimal 300 bp), GC content of 20–60%, and PRIMER_MAX_HAIRPIN_TH = 24. All SSR sequences were also used as a repeat library (option PRIMER_MISPRIMING_LIBRARY) to avoid primer design within the SSRs. Primers with low complexity regions were discarded.

### Analysis of genes involved in fruit ripening

As a proof of concept, our gene-centered SSR map were used to identify SSR markers for genes potentially involved in cell wall remodeling, transcriptional regulation and hormone signaling. Genes with differential expression in tomato ripening fruits (determined by RNA-Seq) [28] were used to identify homologous genes in papaya. Fifty-three cell wall and 222 transcription/ethylene proteins were used as BLASTP queries to search the papaya predicted proteins with the following criteria: E-value ≤1e–30, similarity of at least 50%, query and hit coverage of at least 75%. Tomato protein sequences and annotations release ITAG2.3 were downloaded from the Sol Genomics Network FTP [29] (ftp://ftp.solgenomics.net/).

### SSR screening and polymorphism survey

The papaya genotypes Sekati and JS12 were used for screening polymorphic genic SSRs. Total genomic DNA was extracted from young leaves according to the CBAB method [30]. A total of 73 primer pairs comprising ∼8 genic SSR regions per chromosome were selected. PCR amplifications were performed in 15 µL reaction, containing 10 ng DNA, 10 mM Tris-HCl, pH 8.3, 50 mM KCl, 2 mM MgCl_2_, 100 µM dNTPs, 0.2 µM of each primer, and 1 U Taq DNA polymerase. PCR cycling was performed in an Eppendorf thermal cycler, according to the following profile: 4 min of denaturation at 94°C, 35 amplification cycles (94°C at 30 s, 58°C at 1 min, 72°C at 3 min), followed by a final extension of 7 min at 72°C. In 4 of these cases, the primer annealing temperature was set to 65°C. Amplified products were separated in a 4% agarose gel Metaphor, stained by GelRedTM/Blue Juice mixture (1∶1) and visualized through the MiniBis Pro photodocumentation system (DNR Bio-Imaging Systems Ltd., Jerusalem, Israel).

### Data Access and Retrieval

Information regarding SSR identifiers, genomic coordinates, motif sequence, period, size and exponent, genomic location (i.e. exon, intron, exon-intron, intergenic), linkage group, and gene annotations are fully available in two user-friendly spreadsheets (Table S1, Table S2).

## Results and Discussion

### SSR classification and genomic positioning

We identified 160,318 SSRs with a density of 656 SSR/Mb in the most recent version of the papaya genome (see methods for details). Previous studies of papaya SSRs reported densities of 1,340 SSR/Mb [31] and 746 SSR/Mb [32]. Such disparities in the number of identified microsatellites are usual among different reports, mainly due to differences in the algorithms, parameter settings, minimal repeat length and redundancy filtering [33]–[35]. Differently from other two previous studies [31], [32], we used the *mreps* algorithm, which was ranked as the best algorithm for repeat detection in a systematic study [35]. Specifically, *mreps* does not report all the overlapping repeats, but efficiently retains only the most credible overlapping ones, giving more reproducible and reliable results.

**Figure 1:**
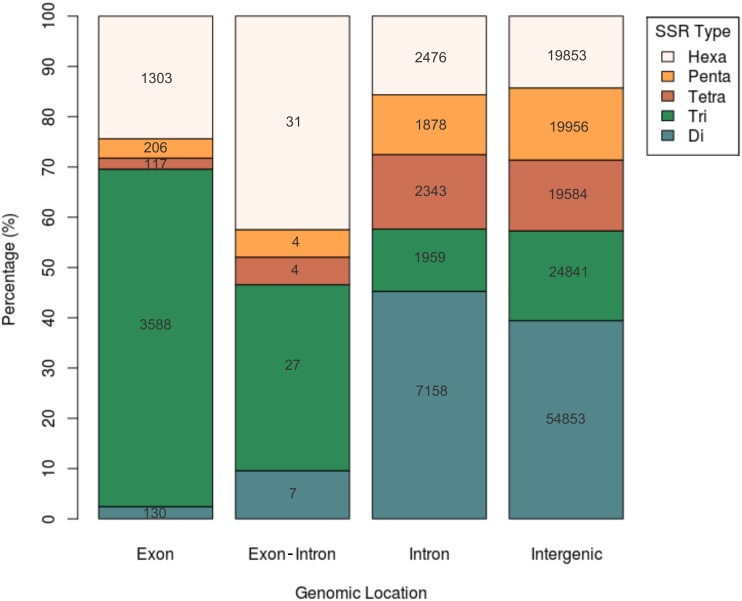
Distribution of SSR type according to genomic location.

We detected 160,318 perfectly matching, non-redundant SSRs. After integrating SSRs and gene coordinates, we found that 36% of the papaya genes (9,992/27,769) have at least one SSR. A total of 21,231 SSRs were identified in genic regions, while 139,087 were intergenic (Figure 1). Because UTR annotations are not available for the papaya genome, SSRs located on these regions were not classified as such. As expected, most SSRs are intergenic (86.8%), followed by intronic (9.9%), exonic (3.3%), and only 73 (0.04%) SSRs in exon-intron boundaries (Table 1). Dinucleotide motifs were abundant in intergenic (39.4%) and intronic regions (45.3%), while tri- to hexanucleotides were uniformly distributed in such regions. On the other hand, exons and exon-intron boundaries were enriched in tri- (67.1% and 37%) and hexanucleotides (24.4% and 42.5%), which is expected due to the selective pressure against frameshift mutations in coding regions.

**Table 1:** Number of simple sequence repeats by genomic location.

	SSR type					
Genomic location	Di	Tri	Tetra	Penta	Hexa	Total
Exon	130	3588	117	206	1303	5344
Exon-intron	7	27	4	4	31	73
Intron	7158	1959	2343	1878	2476	15814
Intergenic	54853	24841	19584	19956	19853	139087
**Total**	**62148**	**30415**	**22048**	**22044**	**23663**	**160318**

Sequence AT/TA was the most common dinucleotide motif (69.9%) in the papaya genome, corroborating previous results obtained from whole-genome shotgun sequences (WGS) and BAC End Sequences (BES) [7], [31]. Nevertheless, when considering genomic locations, this motif was enriched only in intronic (63.6%) and intergenic (70.9%) regions, whereas AG/GA/CT/TC motifs were predominant in exons (88.5%) and exon-intron boundaries (85.7%) (Table 2). Among trinucleotide motifs, AAT/ATA/TAA/ATT/TAT/TTA has been described as most prevalent in papaya genome [7], [31]. Although AAT/TTA sequence was also frequent (47%) in our study, it is mainly located in introns (46.6%) and intergenic (53.3%) regions. Conversely, the second most predominant trinucleotide motif, AAG/AGA/GAA/CTT/TCT/TTC, was more evenly distributed in exons (34.4%), exon-intron boundaries (25.9%), introns (27.9%), and intergenic (21.7%) locations in genomic context. Coherently to a previous study [31], dinucleotides CG/GC and trinucleotides CCG/GGC were rarely found (0.1% and 1.2% respectively) here.

**Table 2:** Distribution of SSRs by genomic location.

Motif	Exon		Exon-intron		Intron		Intergenic		Total	
	# SSRs	Percent(%)	# SSRs	Percent(%)	# SSRs	Percent(%)	# SSRs	Percent(%)	# SSRs	Percent(%)
**Di-**	130	2.4	7	9.6	7158	45.3	54853	39.4	62148	38.8
AT/TA	6	4.6	0	0.0	4554	63.6	38906	70.9	43466	69.9
AC/CA/GT/TG	8	6.2	1	14.3	1049	14.7	7307	13.3	8365	13.5
AG/GA/CT/TC	115	88.5	6	85.7	1546	21.6	8584	15.6	10251	16.5
CG/GC	1	0.8	0	0.0	9	0.1	56	0.1	66	0.1
**Tri-**	3588	67.1	27	37.0	1959	12.4	24841	17.9	30415	19.0
AAC/ACA/CAA/GTT/TGT/TTG	153	4.3	0	0.0	111	5.7	728	2.9	992	3.3
AAG/AGA/GAA/CTT/TCT/TTC	1236	34.4	7	25.9	546	27.9	5387	21.7	7176	23.6
AAT/ATA/TAA/ATT/TAT/TTA	78	2.2	0	0.0	913	46.6	13250	53.3	14241	46.8
GGA/GAG/AGG/TCC/CTC/CCT	406	11.3	3	11.1	44	2.2	537	2.2	990	3.3
GGC/GCG/CGG/GCC/CGC/CCG	102	2.8	1	3.7	10	0.5	260	1.0	373	1.2
GGT/GTG/TGG/ACC/CAC/CCA	316	8.8	7	25.9	49	2.5	350	1.4	722	2.4
ACG/CGA/GAC/CGT/TCG/GTC	89	2.5	2	7.4	7	0.4	107	0.4	205	0.7
ACT/CTA/TAC/AGT/TAG/GTA	225	6.3	1	3.7	88	4.5	2326	9.4	2640	8.7
AGC/GCA/CAG/GCT/TGC/CTG	372	10.4	4	14.8	26	1.3	278	1.1	680	2.2
ATC/TCA/CAT/GAT/TGA/ATG	611	17.0	2	7.4	165	8.4	1618	6.5	2396	7.9
**Tetra-**	117	2.2	4	5.5	2343	14.8	19584	14.1	22048	13.8
**Penta-**	206	3.9	4	5.5	1878	11.9	19956	14.3	22044	13.8
**Hexa-**	1303	24.4	31	42.5	2476	15.7	19853	14.3	23663	14.8
**Total**	**5344**	**100.0**	**73**	**100.0**	**15814**	**100.0**	**139087**	**100.0**	**160318**	**100.0**

**Figure 2:**
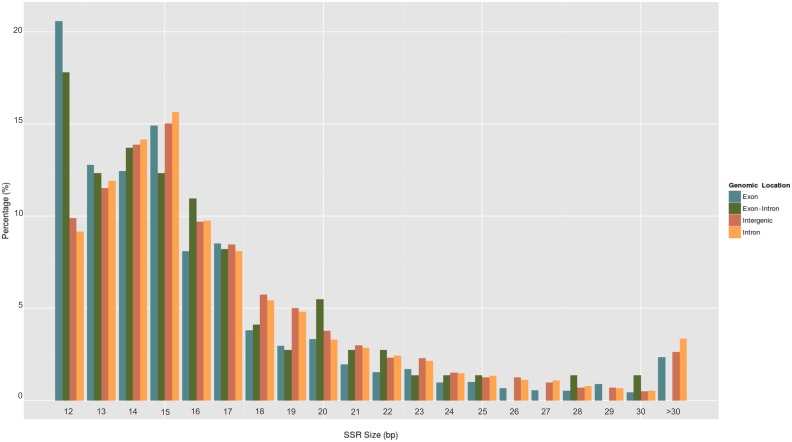
Percentage frequency of SSR according to SSR size.

Based on repeat length, papaya SSRs were defined as class I (≥20 nucleotides) and class II (between 12–19 nucleotides) (Figure 2). SSR lengths ranged from: 12–82 bases in exons; 12–30 bases in exon-intron boundaries; 12–201 bases in introns and 12–155 bases in intergenic regions. Most SSRs were classified as class II (79.0%–84.1%), regardless of their genomic location (Table 3). However, 24,234 primer pairs were designed for class I SSRs (Table S3). Since these longer sequences are typically hypervariable and more likely to be polymorphic, they are the preferable choice as molecular markers for diversity studies.

**Table 3:** Distribution of Class I and Class II SSRs in different genomic regions.

		Exon		Exon-Intron		Intron		Intergenic	
	SSR size (bp)	# SSRs	Percent (%)	# SSRs	Percent (%)	# SSRs	Percent (%)	# SSRs	Percent (%)
**Class II**	12	1100	20.6	13	17.8	1450	9.2	13749	9.9
	13	683	12.8	9	12.3	1883	11.9	16031	11.5
	14	665	12.4	10	13.7	2237	14.1	19308	13.9
	15	796	14.9	9	12.3	2474	15.6	20879	15.0
	16	433	8.1	8	11.0	1542	9.8	13481	9.7
	17	456	8.5	6	8.2	1280	8.1	11760	8.5
	18	203	3.8	3	4.1	861	5.4	7970	5.7
	19	158	3.0	2	2.7	763	4.8	6957	5.0
	**Subtotal**	**4494**	84.1	**60**	82.2	**12490**	79.0	**110135**	79.2
**Class I**	20	178	3.3	4	5.5	522	3.3	5275	3.8
	21	104	1.9	2	2.7	449	2.8	4145	3.0
	22	81	1.5	2	2.7	382	2.4	3223	2.3
	23	91	1.7	1	1.4	340	2.1	3174	2.3
	24	52	1.0	1	1.4	236	1.5	2095	1.5
	25	54	1.0	1	1.4	213	1.3	1741	1.3
	26	36	0.7	0	0.0	176	1.1	1725	1.2
	27	30	0.6	0	0.0	169	1.1	1340	1.0
	28	28	0.5	1	1.4	121	0.8	953	0.7
	29	47	0.9	0	0.0	104	0.7	953	0.7
	30	24	0.4	1	1.4	80	0.5	686	0.5
	>30	125	2.3	0	0.0	532	3.4	3642	2.6
	**Subtotal**	**850**	**15.9**	**13**	**17.8**	**3324**	**21.0**	**28952**	**20.8**
**Total**		**5344**	**100.0**	**73**	**100.0**	**15814**	**100.0**	**139087**	**100.0**

All SSRs were assigned to chromosomes according to their scaffold or contig localization. A total of 116,453 SSRs (72.6%) could be mapped to one of the nine papaya linkage groups. The number of SSRs in each chromosome ranged from 10,566 (6.6%) in LG7 to 14,773 (9.2%) in LG9. The proportion of SSR types and motifs among different chromosomes was similar to the overall genomic distribution (Table S4). The proportion of SSR genomic locations in each chromosome was higher in intergenic regions, followed by introns, exons and exon-intron junctions. There was no bias for SSR types and SSR genomic locations among the chromosomes (Table S5), which is highly desirable for researchers aiming to develop a collection of polymorphic markers for genetic studies.

### Design of high-quality primer pairs for papaya SSRs

Aiming to provide a comprehensive source of SSRs to be used in marker-assisted selection and population genetics studies, all 21,231 genic and 139,087 intergenic SSRs were submitted to primer design. All primer pairs were optimized for the same PCR conditions (see methods for details). In a preliminary analysis, 20,659 (97.3%) and 118,831 (85.4%) primer pairs were respectively designed for genic and intergenic SSRs using Primer3 with default parameters. Manual inspection of results revealed a significant number of primers containing repeated or low complexity sequences. Therefore, we decided to adopt more stringent criteria for primer design: 1) Primers within repetitive sequences were removed; 2) Primers in soft-masked low complexity 3′ end regions were not allowed (PRIMER_LOWERCASE_MASKING = 1); and 3) A parameter to minimize hairpin formation (PRIMER_MAX_HAIRPIN_TH = 24) was employed. By using this stringent parameterization, we obtained a much more reliable primer set, although the overall success rate of primer design dropped to 71%. A total of 18,925 primer pairs were successfully designed for 89.1% and 67.9% of all genic and intergenic SSR sequences, respectively (Table 4). Although such stringent settings prevented primer design for 8% genic and 17% intergenic SSRs, this methodology resulted in an extensive and more reliable list of primer pairs designed for distinct SSR types, genomic locations and chromosome linkage groups (Table S1, Table S2).

**Table 4:** Number of successfully designed primer pairs for SSR type and linkage group.

SSRType	LG1		LG2		LG3		LG4		LG5		LG6		LG7		LG8+LG10^a^		LG9		Un^b^		Total	
	#	%	#	%	#	%	#	%	#	%	#	%	#	%	#	%	#	%	#	%	#	%
**2**	3669	41.3	4254	41.3	4212	40.4	3433	40.9	3154	39.5	4429	41.2	3221	41.7	3988	41.7	4446	41.1	10200	35.6	45008	39.7
**3**	1645	18.5	1922	18.7	1892	18.2	1565	18.6	1414	17.7	1871	17.4	1307	16.9	1639	17.1	1937	17.9	5483	19.2	20677	18.2
**4**	1233	13.9	1357	13.2	1351	13.0	1163	13.8	1068	13.4	1436	13.4	1003	13.0	1242	13.0	1458	13.5	3780	13.2	15093	13.3
**5**	1066	12.0	1271	12.4	1430	13.7	1048	12.5	1114	14.0	1342	12.5	1029	13.3	1252	13.1	1347	12.4	4539	15.9	15441	13.6
**6**	1264	14.2	1486	14.4	1531	14.7	1193	14.2	1225	15.4	1659	15.5	1158	15.0	1449	15.1	1639	15.1	4621	16.1	17227	15.2
**Total**	**8877**	**100.0**	**10290**	**100.0**	**10416**	**100.0**	**8402**	**100.0**	**7975**	**100.0**	**10737**	**100.0**	**7718**	**100.0**	**9570**	**100.0**	**10827**	**100.0**	**28623**	**100.0**	**113446**	**100.0**

LG represents the Linkage Groups associated to the nine papaya chromosomes.

a LG8 and LG10 are associated to the same chromosome.

b SSRs located in scaffolds/contigs were classified as unplaced.

Designed primers were uniformly distributed among SSR types (Table 4) and chromosomes (Table 5). Regarding SSR type, most of the 113,446 primer pairs were designed for dinucleotide repeats (39.7%), followed by tri- (18.2%), hexa- (15.2%), penta- (13.6%) and tetranucleotide repeats (13.3%) (Table 4). As expected, the majority of exonic SSRs with designed primers are composed of trinucleotide repeats (Table S6).

**Table 5:** Number of successfully designed primer pairs for each genomic context and linkage group.

Genomic location	LG1		LG2		LG3		LG4		LG5		LG6		LG7		LG8+LG10^a^		LG9		Un^b^		Total	
	#	%	#	%	#	%	#	%	#	%	#	%	#	%	#	%	#	%	#	%	#	%
**Exon**	443	5.0	576	5.6	466	4.5	385	4.6	349	4.4	540	5.0	316	4.1	414	4.3	520	4.8	693	2.4	4702	4.1
**Exon-intron**	10	0.1	7	0.1	9	0.1	5	0.1	8	0.1	8	0.1	5	0.1	4	0.0	2	0.0	5	0.0	63	0.1
**Intron**	1457	16.4	1468	14.3	1442	13.8	1231	14.7	1140	14.3	1589	14.8	1029	13.3	1346	14.1	1440	13.3	2018	7.1	14160	12.5
**Intergenic**	6967	78.5	8239	80.1	8499	81.6	6781	80.7	6478	81.2	8600	80.1	6368	82.5	7806	81.6	8865	81.9	25907	90.5	94521	83.3
**Total**	**8877**	**100.0**	**10290**	**100.0**	**10416**	**100.0**	**8402**	**100.0**	**7975**	**100.0**	**10737**	**100.0**	**7718**	**100.0**	**9570**	**100.0**	**10827**	**100.0**	**28623**	**100.0**	**113446**	**100.0**

LG represents the Linkage Groups associated to the nine papaya chromosomes.

a LG8 and LG10 are associated to the same chromosome.

b SSRs located in scaffolds/contigs were classified as unplaced.

### Analysis of SSRs located in genes related to fruit ripening

Next, we aimed to use our SSR atlas to find genes related to fruit ripening, a trait of high agronomical interest in papaya. To achieve this goal, tomato gene expression and tomato/papaya orthology data were integrated. Tomato is the papaya’s closest climacteric fleshy fruit with available genome-wide gene expression data [28]. Sato et al. reported significant differential expression of 53 cell wall and 222 transcription factors (TFs)/ethylene-related genes during fruit ripening [28]. Based on BLASTP searches (see methods for details), 175 cell wall and 319 transcription factor orthologous genes were found in papaya (Table S7, Table S8). Our atlas include SSRs with primer pairs for 113 cell wall-related (257 SSRs, 40 exonic and 217 intronic; 2.3 SSRs/gene) and 187 TF/ethylene-related genes (528 SSRs, 127 exonic and 400 intronic; 2.8 SSRs/gene). These two groups comprise very good candidate pulp softening and pigmentation control genes [36]. By integrating this information in our gene-centered SSR map, we provide an unprecedented list of markers for studying the genetic and functional variability of fruit ripening processes.

Fruit ripening is a developmental process characterized by remarkable changes related to flavor, sugar metabolism, color, aroma, texture, softening and nutritional content [37]. These metabolic and physical alterations are driven by genetically coordinated expression profiles in several metabolic pathways, such as cell wall disassembly, sugar hydrolysis, ethylene biosynthesis and pigmentation. Using KEGG Orthology (KO) we identified ripening-related pathways for cell wall genes and TFs harboring SSRs with primer pairs. Nine entries for cell wall genes were found in KO00050 - Starch and sucrose metabolism (5) and KO00040– Pentose and glucuronate interconversions (4). During climacteric fruit ripening, respiration rate increases and a series of enzymes degrade starch and synthesize sucrose (e.g. starch phosphorylase and sucrose synthase) [38]. The carboxylic acid glucuronate is the precursor of pectin, one of the main components of plant cell wall [39]. Pectin levels decrease during fruit maturation [39] typically due to the increased expression of pectin lyases [28]. However, since pectin lyases and methyltransferases are repressed during papaya ripening, pectin solubilization is probably catalyzed by polygalacturonases [40].

Among TFs/ethylene-related genes, 24 entries were found, mostly from the category KO04075 (Plant hormone signal transduction; 8 genes), such as auxin-responsive protein, ethylene receptor and responsive transcription factor 1 and ABA-responsive element binding factor. The phytohormone auxin plays important roles in fruit growth, regulating cell division, differentiation, lateral root formation and embryogenesis [41]. Particularly, genes involved in signaling and auxin response factors were up-regulated during papaya [40] and peach [42] ripening. In addition, auxin can stimulate ethylene biosynthesis via transcription of acetyl-coenzyme A synthetase genes [42]. In turn, the involvement of ethylene response factor in climacteric fruit ripening is well-established and this regulator determines, for example, fruit firmness reduction and defense response to pathogens after ripe [36]. By aggregating these annotations, our atlas provides a rich resource from which the scientific community can rapidly draw genes and SSRs with designed primer pairs for genetic studies.

### SSR screening and polymorphism survey

A 100% PCR amplification rate was achieved for the 73 genic SSRs (16 exonic and 57 intronic), allowing the identification of 19 polymorphic alleles (26%) (Table S9). Twenty five of such genes are orthologs of tomato genes with differential expression during fruit ripening (Table S7, Table S8). Among the polymorphic alleles we found 5 cell wall and 2 transcription factors/ethylene-related genes. Polymorphisms were also detected in genes from the cellulose synthase, pectin lyase-like and ethylene response factor families/superfamilies. Taken together, these results not only validate the use of our atlas as an efficient tool in papaya breeding projects, but also stimulate additional genetic and biochemical studies to detail the functions these polymorphic genes in papaya, as they may be useful in the production of fruits with increased shelf life.

## Conclusion

Non-coding SSRs near genes or inside introns can directly affect gene expression [21], [43]–[45]. On the other hand, SSRs within exons often result in amino acid changes that may affect protein function. For example, a study using genic SSRs derived from candidate genes involved in wood formation identified two SSR markers (one in coding and the other in non-coding region) explaining 13.5% of the lignin content variation in Chinese white poplar [46]. These results demonstrate the power of genic markers to identify genotype-to-phenotype associations and make these markers very useful in genetic improvement of desired characteristics.

In the present work we surveyed the papaya genome for the presence of perfect, non-redundant SSRs. We analyzed the distribution of SSR locations (exon, exon-intron, intron, intergenic) and established a comprehensive atlas of SSR markers with SSR type, motif sequence, SSR size, genomic location (exon, intron or intergenic), linkage group location and gene-centered information (gene annotation and GO assignments). The resource reported here is fully accessible through our supplementary material (Table S1, Table S2), allowing plant breeders and researchers to easily choose gene-centered markers to test their association with biological processes or phenotypes of agronomic interest. Moreover, we achieved a 100% PCR amplification rate during a genetic survey of 73 SSR markers, supporting the high quality of the predicted SSRs and designed primers. The atlas developed in this study will certainly serve as a toolbox to assist and improve the efficiency of marker-assisted selection in papaya breeding and population genetic studies.

## Supporting Information

Table S1 **Catalog of gene-centered SSR markers within genic regions (exon, exon-intron, intron).**
(ZIP)

Table S2 **Catalog of SSR markers within intergenic regions.**
(ZIP)

Table S3 **Class I SSR markers.**
(ZIP)

Table S4 **Distribution of SSR motifs by linkage group.**
(XLS)

Table S5 **Distribution of SSR types by linkage group.**
(XLS)

Table S6 **Number of successfully designed primer pairs for each SSR type, genomic location and linkage group.**
(XLS)

Table S7 **SSR markers for cell wall genes.**
(XLS)

Table S8 **SSR markers for transcriptional/ethylene genes.**
(XLS)

Table S9 **Primer pairs used for polymorphism analysis.** Genes related with cell wall metabolism and transcriptional regulation/ethylene signaling genes are highlighted in green and yellow, respectively.
(XLS)
